# Supplementary material for: Crystalline metamaterials for topological properties at subwavelength scales
Source: Nat Commun. 2017 Jul 18;8:16023. doi: 10.1038/ncomms16023 (PMC5520060; doi:10.1038/ncomms16023)
Supplement: Supplementary Information [file ncomms16023-s1.pdf]

Description of Supplementary Files

File Name: Supplementary Information
Description: Supplementary Figures

File Name: Peer Review File

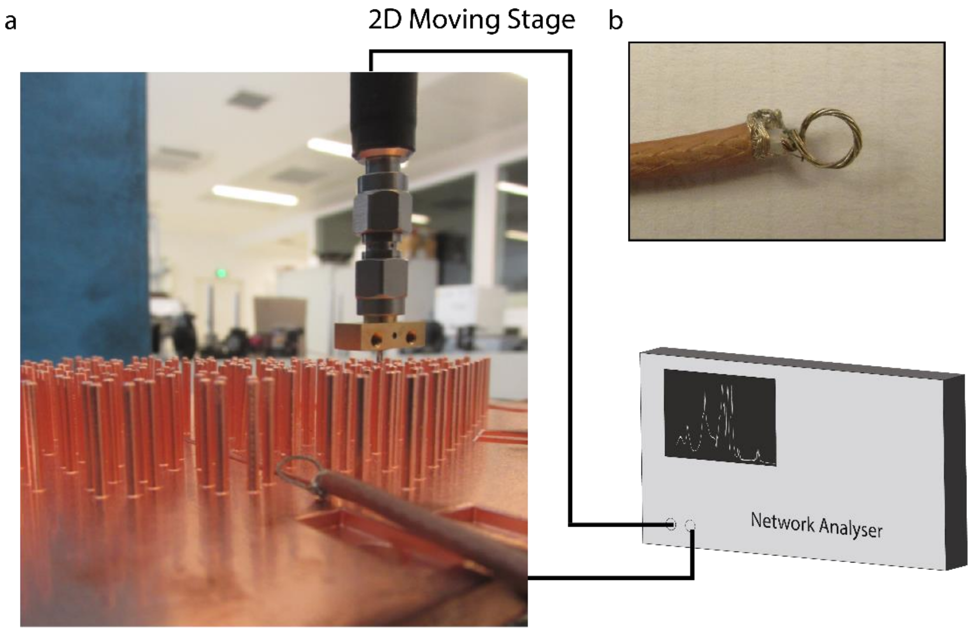

Supplementary Figure 1: ***Experimental setup for the measurement of the electric field maps.*** *We measure the spectrum of the transmission between a small loop antenna (zoom in (**b**)) and a field probe, while scanning the sample right above the wires.*

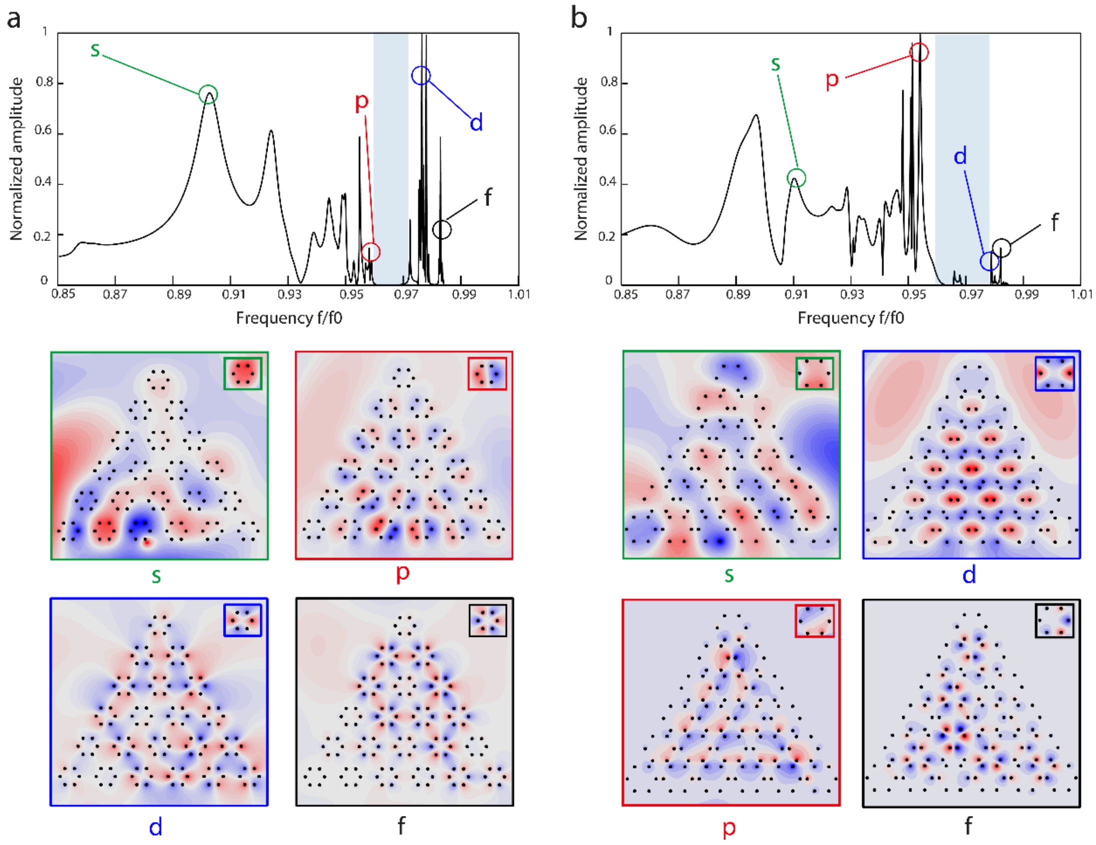

Supplementary Figure 2: ***Semi-analytical modeling of the bulk experiments.*** *(a) Calculated spectrum corresponding to the topologically trivial lattice. (c–f) Semi-analytical electric field maps corresponding to the labeled peaks of the spectrum. (b) Same as a for the topologically non-trivial lattice. The corresponding semi-analytical electric*

field maps are displayed on (g–j). This shows that our coupled-dipole analytical code is used to corroborate our experimental findings and confirm the generality of our results.

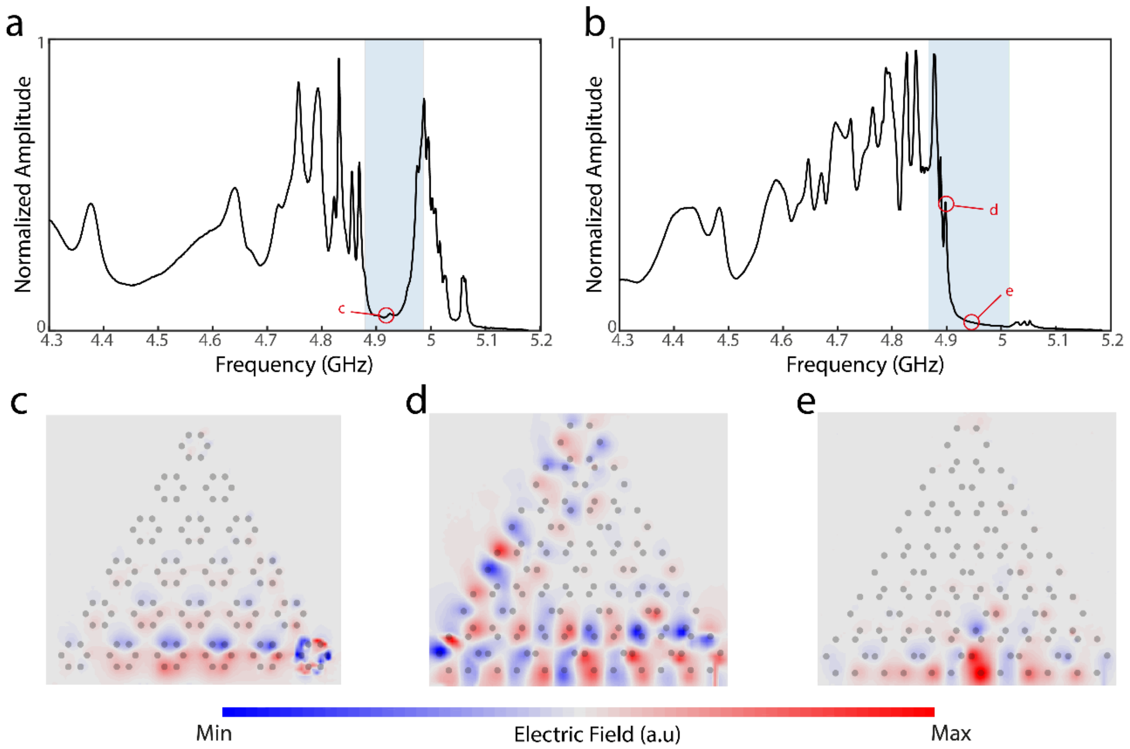

Supplementary Figure 3: ***Experimental study of edge modes with free-space for both trivial and topological samples.*** *Spectra averaged along the trivial sample contour (a) and the topological one (b). Electric field maps measured above the trivial sample (c) and the topological one (d,e). Only the topological sample supports wave propagation on its edges with free-space (d).*

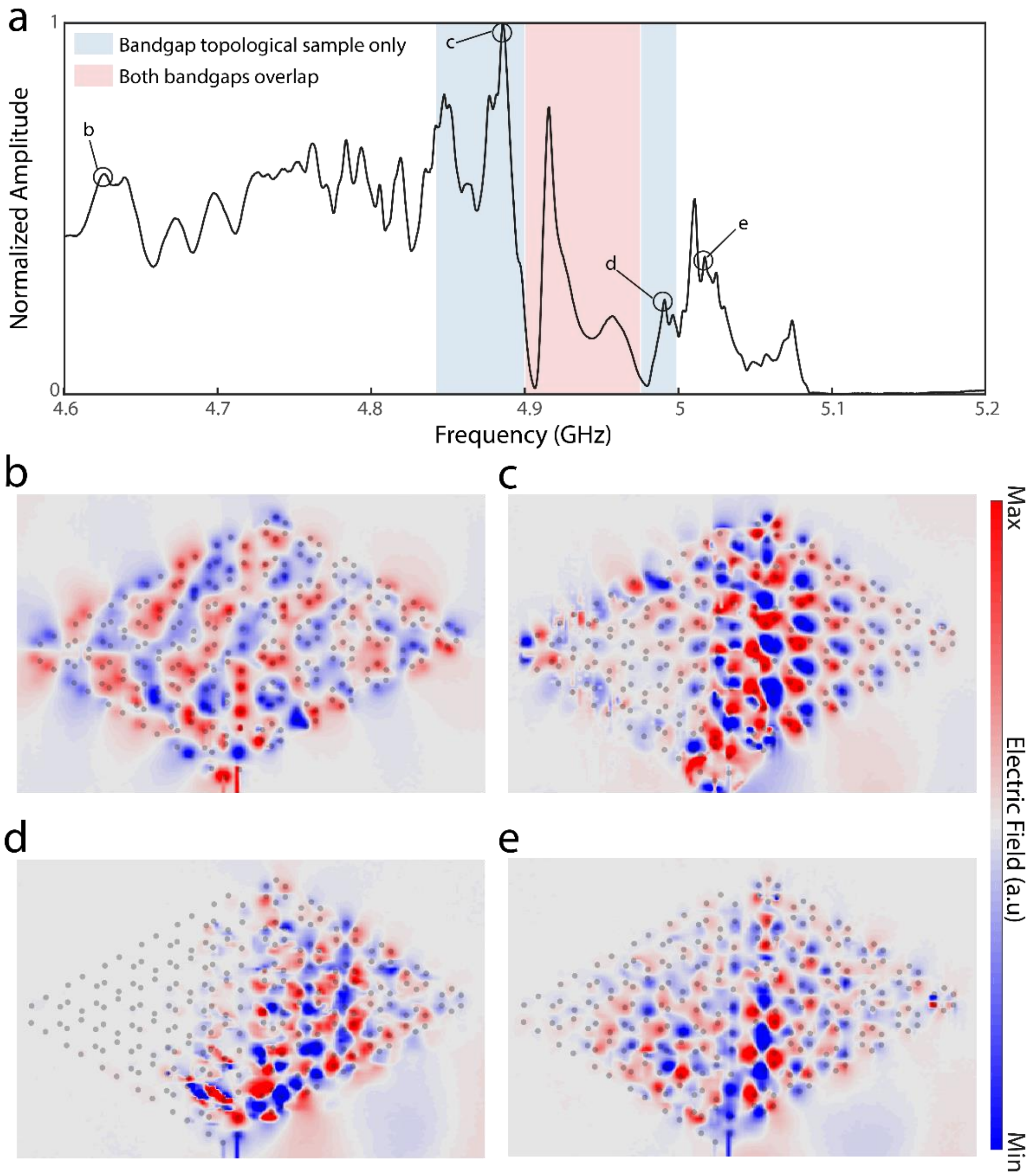

Supplementary Figure 4: ***Experimental study of the interface experiment for frequencies outside the mutual bandgap.*** *(a) Spectrum averaged on an area containing the domain wall between the two media. Electric field maps measured at frequencies inside the bulk of both samples (b,e). Field maps measured for a frequency inside the bandgap of the topological sample only (c,d).*

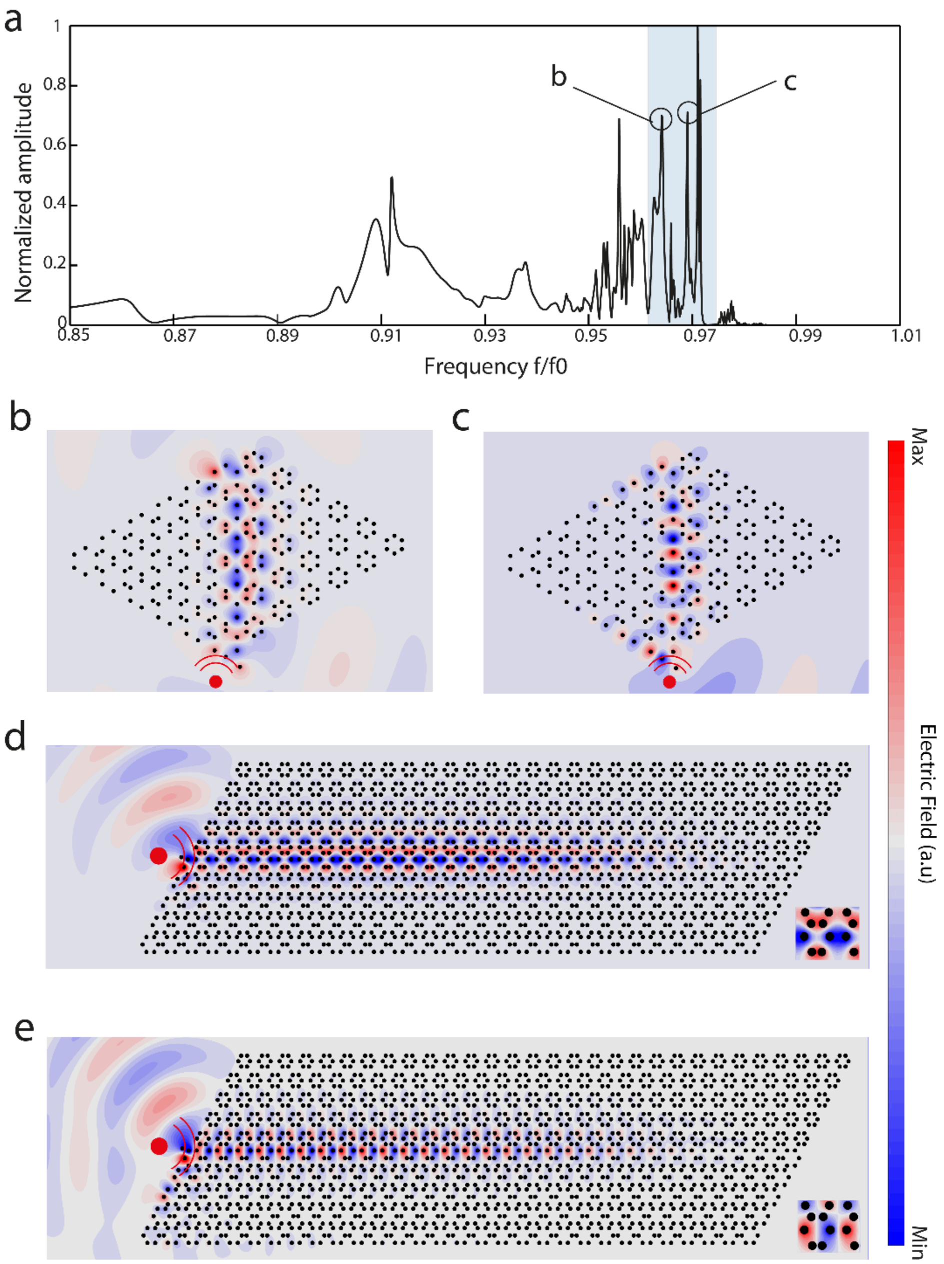

Supplementary Figure 5: ***Semi-analytical simulation of the interface experiment.*** *(a) Transmission spectrum calculated analytically at a point on the interface between the two samples. (b,c) Corresponding modes propagating along the interface. (d,e) Simulation with a Perfectly Matched Layer (PML) at the right of the sample (last 10 crystal columns), highlighting the electric field distribution corresponding to pure edge modes (insets).*

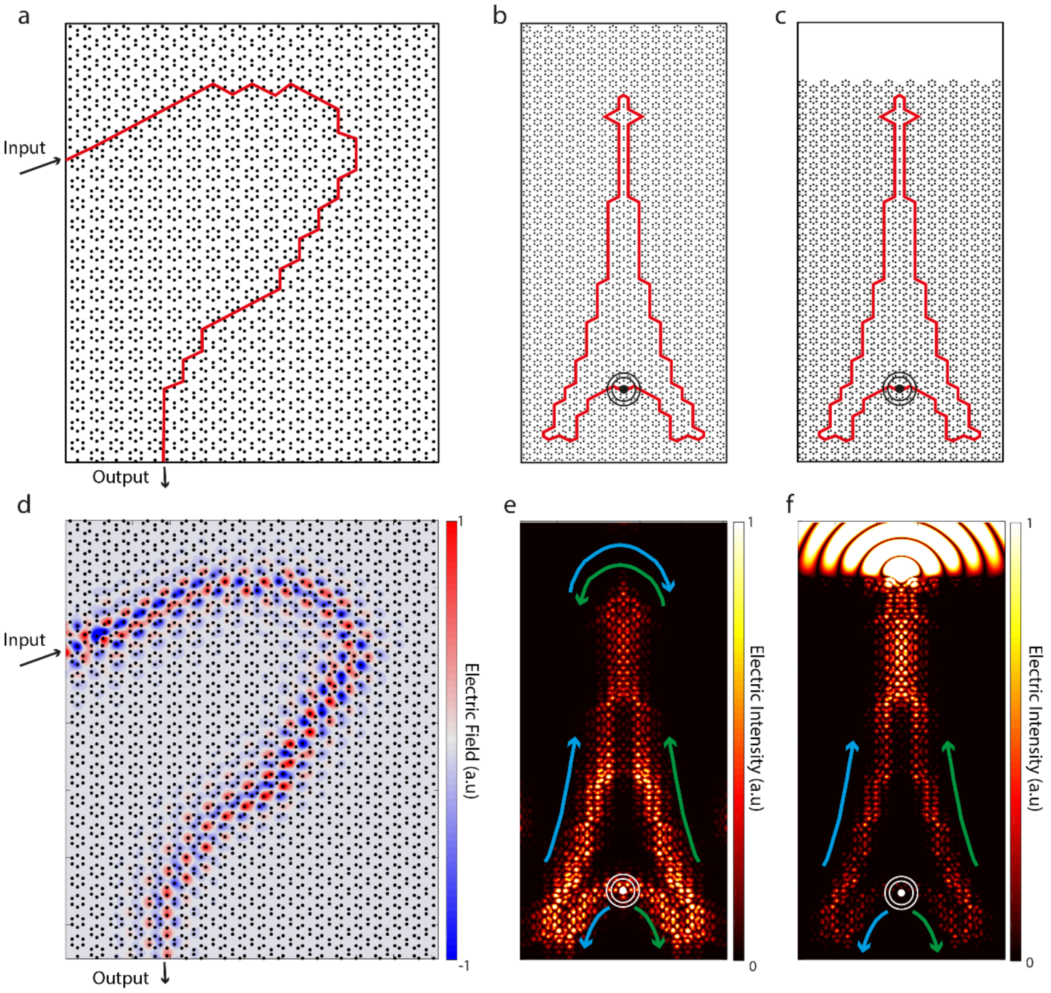

Supplementary Figure 6: ***Numerical study of robust subwavelength propagation along the interface between a trivial and a topological medium.*** *(a) Tortuous path (red line) between topological and trivial medium. (b) Closed cavity (red line) shaped as the Eiffel tower. The source is set at the bottom of its first floor. (c) Same as b but the top part of the trivial medium is replaced by free-space. (d), Electric field map corresponding to a, demonstrating robust subwavelength propagation of the mode along the domain wall. (e) Electric intensity map corresponding to (b). Waves are emitted by the source and propagate in both directions (green and blue arrows), exciting the subwavelength cavity. (f) Electric intensity map corresponding to c showing the good matching to free-space, and good radiation at the top of the Eiffel tower.*
